# Giant quantum oscillations in thermal transport in low-density metals via electron absorption of phonons

**DOI:** 10.1073/pnas.2408546122

**Published:** 2025-03-05

**Authors:** Baptiste Bermond, Rafał Wawrzyńczak, Sergei Zherlitsyn, Tommy Kotte, Toni Helm, Denis Gorbunov, Genda Gu, Qiang Li, Filip Janasz, Tobias Meng, Fabian Menges, Claudia Felser, Joachim Wosnitza, Adolfo Grushin, David Carpentier, Johannes Gooth, Stanisław Gałeski

**Affiliations:** ^a^École normale supérieure de Lyon, CNRS, Laboratoire de Physique, Lyon F-69342, France; ^b^Max Planck Institute for Chemical Physics of Solids, Dresden 01187, Germany; ^c^Hochfeld-Magnetlabor Dresden-European Magnetic Field Laboratory and Würzburg-Dresden Cluster of Excellence ct.qmat, Helmholtz-Zentrum Dresden-Rossendorf, Dresden 01328, Germany; ^d^Department of Condensed Matter Physics and Materials Science, Brookhaven National Laboratory, Upton, NY 500; ^e^Department of Physics and Astronomy, Stony Brook University, Stony Brook, NY 11794-3800; ^f^Department of Engineering, Faculty of Science, Medicine and Technology, University of Luxembourg, Luxembourg 1359, Luxembourg; ^g^Institute for Theoretical Physics, Würzburg-Dresden Cluster of Excellence ct.qmat, Technische Universität Dresden, Dresden 01069, Germany; ^h^Institut für Festkörper-und Materialphysik and Würzburg-Dresden Cluster of Excellence ct.qmat, Technische Universität Dresden, Dresden 01062, Germany; ^i^Univ. Grenoble Alpes, CNRS, Institut polytechnique de Grenoble, Institut Néel, Grenoble 38000, France; ^j^Physikalisches Institut, Universität Bonn, Bonn 53115, Germany

**Keywords:** Dirac semimetal, thermal transport, Wiedemann–Franz law, quantum limit, Landau levels

## Abstract

Oscillations of conductance observed in strong magnetic fields are a striking manifestation of the quantum dynamics of charge carriers in solids. The large charge carrier density in typical metals sets the scale of oscillations in both electrical and thermal conductivity, which characterize the Fermi surface. In semimetals, thermal transport at low-charge carrier density is expected to be phonon dominated, yet several experiments observe giant quantum oscillations in thermal transport. This raises the question of whether there is an overarching mechanism leading to sizable oscillations that survives in phonon-dominated semimetals. In this work, we show that such a mechanism exists. It relies on the peculiar phase-space allowed for phonon scattering by electrons when only a few Landau levels are filled. Our measurements on the Dirac semimetal ZrTe_5_ support this counterintuitive mechanism through observation of pronounced thermal quantum oscillations, since they occur in similar magnitude and phase in directions parallel and transverse to the magnetic field. Our phase-space argument applies to all low-density semimetals, topological or not, including graphene and bismuth. Our work illustrates that phonon absorption can be leveraged to reveal degrees of freedom through their imprint on longitudinal thermal transport.

## Significance

In metals thermal conductivity is proportional to electrical conductivity and typically of little interest. Here, we have observed giant quantum oscillations in thermal conductivity of the Dirac semimetal ZrTe_5_, four orders of magnitude larger than expected, even though thermal transport at zero-field is dominated by phonons, which are neutral and thus insensitive to magnetic fields. We identify a generic mechanism for an enhanced absorption of phonons by electrons for metals in quantizing magnetic fields, leading to appearance of quantum oscillations in transport properties of phonons. Our results demonstrate that phonon absorption can be leveraged to reveal other degrees of freedom through their imprint on longitudinal thermal transport of phonons, even if those degrees of freedom have negligible contribution to thermal conductivity.

Magnetic quantum oscillations are a captivating phenomenon revealing the quantum dynamics of electrons in solids. (1, 2). They occur due to the quantization of energy levels of electrons when subjected to an external magnetic field, known as Landau levels (LLs). The electronic properties of a solid undergo changes as the number of filled Landau levels varies with the magnetic field, manifesting as oscillations in various physical observables. Among those, the Shubnikov–de Haas oscillations of electrical resistivity (3–5) and the de Haas-van Alphen oscillations of the magnetic susceptibility (6) have become wide-spread tools to characterize the Fermi liquid-like behavior of electrons in crystals.

In contrast, the more challenging measurements of quantum oscillations in thermal conductivity are typically expected to bring little additional insight beyond Shubnikov–de Haas oscillations. The reason is that, in common metals, the electronic contribution to thermal transport is expected to satisfy the Wiedemann–Franz (WF) law, which relates DC thermal conductivity κ to the electrical one σ as $\kappa =L_{0}T\sigma$, where $L_{0}=\frac{\pi^{3}}{3}{\left(k_{B}/e\right)}^{2}$ is the Lorentz number and T the temperature. The proportionality between σ and κ reflects the fact that the same electronic degrees of freedom that carry charge also carry heat, resulting in similar quantum oscillations.

The situation is more involved in semimetals, for which the density of charge carriers can be several orders of magnitude smaller than in common metals such as copper. Whether the thermal transport is dominated by electrons, such as in metals, or by phonons, such asUpper Left Panel in insulators, is a subtle question. On one hand, the thermal conductivity of bismuth is dominated by the phononic contribution with a characteristic $T^{3}$ behavior at low temperatures (7–9). On the other hand, recent magneto-thermal transport experiments on a range of Weyl semimetals have displayed large enhancements of the Lorenz ratio interpreted as a nontrivial electronic contribution.

In the Weyl semimetals, NbP (10) and TaAs (11) measurements of the longitudinal magneto-thermal conductivity have revealed the presence of quantum oscillations more than two orders of magnitude larger than expected based on the WF law. Due to the weak field dependence of phonon attenuation and presence of well-defined chiral charge carriers, it was suggested that the enhancement of magneto-thermal oscillations could originate from a novel collective excitation of the Fermi sea of chiral fermions, known as chiral zero sound (CZS). Interestingly, experimental work focusing on the transverse magnetothermal conductivity has also detected giant quantum oscillations. Here, the apparent WF violation was interpreted as originating from ambipolar conduction accounting for contributions of both electron- and hole-like excitations (12). These experiments seem to discourage a general mechanism that leads to large thermal quantum oscillations applicable to all low-density semimetals.

In this work, we put forward experimental and theoretical evidence of a phonon-electron scattering mechanism that leads to large magneto-thermal quantum-oscillations in all low-density semimetals, even when phonons dominate heat transport. It rests on the peculiar phase-space constraints on the allowed phonon absorption mechanisms by electrons that occur when only a few Landau levels are filled.

As a consequence, magneto-thermal quantum oscillations have similar amplitudes and phase in directions along and perpendicular to the magnetic field. Besides the quantum oscillations, the varying phonon-absorption mechanisms parallel and perpendicular to the magnetic field B lead to different B dependences: a linear-in-B increase of the thermal conductivity along the field, as opposed to a constant field dependence in directions perpendicular to the field.

We experimentally support this mechanism through a study of thermal transport in the Dirac semimetal ZrTe_5_. Owing to its simple band structure, a single low-density band crossing the Fermi energy, we are able to quantitatively rationalize its giant quantum oscillations in thermal resistance, observed both in the longitudinal and transverse directions with respect to the magnetic field. Notably, the heat transport is phonon dominated, as we find a clear $T^{3}$ temperature dependence of the amplitude of quantum oscillations. Because our analysis is based on phase-space arguments, we argue that it is generic to low-density metals, including bismuth and graphene. More broadly, this mechanism seems indispensable to disentangle band-structure effects from strongly correlated phenomena in magneto-thermal quantum oscillations.

## Results

**Fig. 1.**
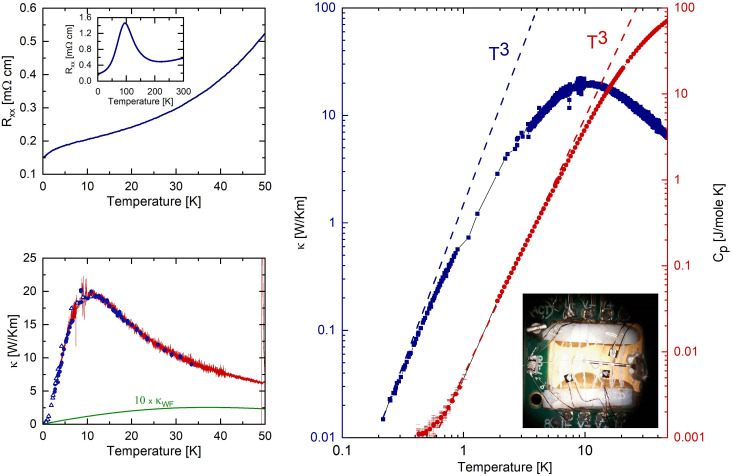
Thermal and electrical transport in ZrTe_5_. (*Upper Left Panel*) Electrical resistivity of ZrTe_5_ measured with a 10 μA current passed along the crystallographic a-axis. (*Lower Left Panel*) Thermal conductivity of ZrTe_5_ measured with a thermal gradient (ΔT<0.1T) applied along the a-axis. The continuous red line represents measurements where temperature was slowly changed and the gradient continuously recorded. Full circles and triangles represent measurements performed in two distinct cooldowns with temperature being stabilized for several minutes before taking the measurements. The solid green line represents an estimate of the thermal conductivity due to the electrons obtained from the Wiedemann–Franz law. (*Right Panel*) Comparison of thermal conductivity (blue points) and specific heat data (red points) and the expected $T^{3}$ dependence (dashed lines). Black hairlines act as a guide to the eye for the data. The *Inset* displays a microscope photograph of one of the custom-build thermal conductivity setups. Cernox thermometers are attached to the sample using a 100 μm silver wire. In this setup, used below 1 K, superconducting TiN wires were used as electrical leads to the thermometers and heater. For measurements at higher temperatures long sections of manganin wire were used as electrical leads for thermal insulation.

In this study, we have selected ZrTe_5_ samples whose electronic properties and Fermi-surface topology have been thoroughly studied (for sample details, see appendix of ref. 13). One challenge in the study of transport properties of topological Weyl and Dirac semimetals lies in their often complex band structure (14). The presence of different charge carrier types and multiple Fermi surface sheets of nontrivial shape often makes comparison between theoretical predictions and transport experiments demanding. One exceptional family of compounds are the pentatellurides ZrTe_5_ and HfTe5. Multiple studies have shown that samples with low charge-carrier densities $n<10^{18}$ cm^-3^, harbor only a single electron-like elliptical Fermi surface at the Γ point comprising only few percent of the Brillouin zone and electron mobilities of the order of 400,000 cm^2^s^-1^V^-1^ (15–20). This makes the pentatellurides one of the simplest Dirac materials with only a single valley of electrons. Indeed, our recent study of electrical and thermoelectric transport, magnetization, and sound propagation measurements has shown good agreement with linear response calculations based on the simple anisotropic Dirac Hamiltonian (13). All measured samples harbor a small charge-carrier density of approx. $1\cdot 10^{17}$ cm^−3^ and a single electron-like Fermi surface located at the Γ point comprising less than 5% of the Brillouin zone (BZ). Thermal-transport data presented in this study has been measured using a homemade thermal-transport setup; see *Inset* of Fig. 1*C* (for details, see *SI Appendix*).

As the first step toward understanding the thermal properties of ZrTe_5_, we have compared its electrical (Fig. 1*A*) with its thermal (Fig. 1*B*) transport properties, when both heat and electrical currents are applied along the crystallographic a-axis. The measured thermal conductivity κ displays a broad maximum at *ca.*10 K reaching the value of ∼20 W/Km, in good agreement with previous reports (21–24). This maximum is attributed to the saturation of the phonons’ mean free path due to scattering from crystal surfaces and large crystal defects such as grain boundaries (9). Comparison of the thermal conductivity with an estimate of the electronic contribution to the thermal transport derived from the WF law reveals, as expected for a low charge-carrier density metal, that the electronic contribution to the overall thermal conductivity is negligible (green line in Fig. 1*B*). This is additionally confirmed by the temperature dependence of κ at low temperatures (Fig. 1*C*) where it follows the canonical $T^{3}$ temperature dependence expected for a thermal conductivity dominated by phonons (9). Interestingly, our high-resolution measurement does not reveal any signatures of phonon hydrodynamics as suggested in ref. 22. In particular, the slope of κ never exceeds the canonical $T^{3}$ temperature dependence.

Similarly, the measured specific heat shown in Fig. 1*C* exhibits a $T^{3}$ dependence down to 500 mK suggesting a dominant role of the phonon degrees of freedom and a saturated mean free path. This is in reasonable agreement with ref. 25, and Shaviv et al. reported a slightly lower exponent (26). At temperatures below 600 mK, a small excess in specific heat is observed. However, due to the exceedingly small specific heat of the samples compared to the calorimeter cell, those data have a significant measurement uncertainty and will be discarded in further analysis.

**Fig. 2.**
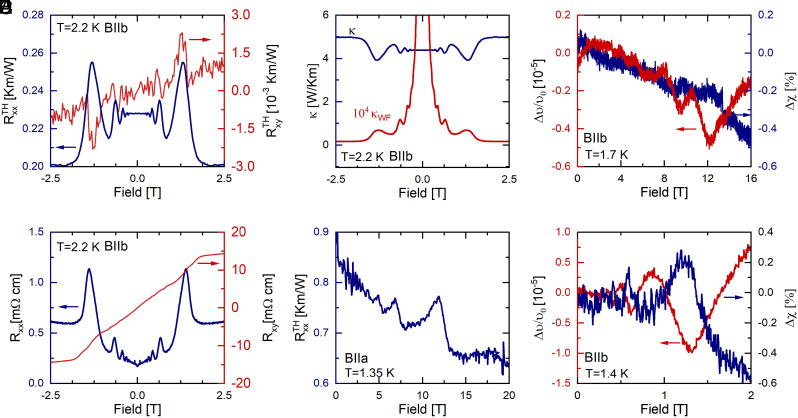
Magnetotransport experiments on ZrTe_5_. (*A*) Symmetric and antisymmetric components of the thermal magneto-resistance tensor of ZrTe_5_ measured at 2.2 K and with magnetic field applied parallel to the crystallographic b-axis and thermal gradient parallel to the a-axis. (*B*) Comparison of transverse thermal conductivity calculated from thermal magneto-resistance measurement (blue) and an estimate based on the Wiedemann–Franz law (red). (*C*) Sound-velocity variation $\Delta v/v_{0}$ (red) and echo transmission amplitude (blue) of a longitudinal sound mode propagating along the a-axis as a function of magnetic field applied along the a-axis. (*D*) Symmetric and antisymmetric components of the magneto-resistance tensor of ZrTe_5_ measured at 2.2 K and with magnetic field applied parallel to the crystallographic b-axis and electric current parallel to the a-axis. (*E*) Thermal magneto-resistance of ZrTe_5_ measured at 1.3 K and with magnetic field and thermal gradient applied parallel to the crystallographic a-axis. (*F*) Sound-velocity variation $\Delta v/v_{0}$ (red) and echo transmission amplitude (blue) of a longitudinal sound mode propagating along the a-axis as a function of magnetic field applied along the b-axis.

Although the thermal transport of ZrTe_5_ appears rather conventional up to this point, further investigation of its field dependence unveils rather uncommon features. Fig. 2*A* shows the magneto-thermal resistance and Righi-Leduc (thermal Hall) effect with the magnetic field applied along the crystallographic b-axis measured at 2.2 K. Inspection of the data reveals a negligibly small thermal Hall contribution to transport, on the edge of measurement sensitivity (red curve). In contrast, the longitudinal component of the thermal magneto-resistance exhibits giant oscillations matching the Shubnikov de-Haas oscillations observed in electrical resistance, Fig. 2*D*, measured on the same sample. The close resemblance of both effects suggests a common electronic origin of these oscillations, related to the Landau quantization of electronic orbits. This, however, is in stark contrast with the magnitude of the effect: The amplitude of the last oscillation amounts to almost 20% of the total thermal resistance, even though the electronic contribution to thermal transport at zero field, estimated based on the WF law, is less than 0.8% of the total thermal conductivity. Indeed, comparison of the calculated thermal magneto-conductivity with an estimate based on the WF law, Fig. 2*B*, reveals that the amplitude of the measured thermal oscillations is about a factor $10^{4}$ larger than expected from an electronic contribution. In addition, the overall shape of the thermal magneto-conductivity differs significantly from the expected $B^{-1}$ behavior.

Moreover, quantum oscillations of the thermal conductivity of similar magnitude are also visible when both the thermal gradient and magnetic field are parallel to the a-axis, Fig. 2*E*. However, in this case, due to the presence of current jetting (27–29), it is not possible to compare their magnitude with those of the electrical resistance (*SI Appendix* for details). Interestingly, in this measurement configuration, we observe, in addition to quantum oscillations, a negative contribution to the magneto-thermal resistivity (positive magneto-thermal conductivity; see *Discussion* below).

**Fig. 3.**
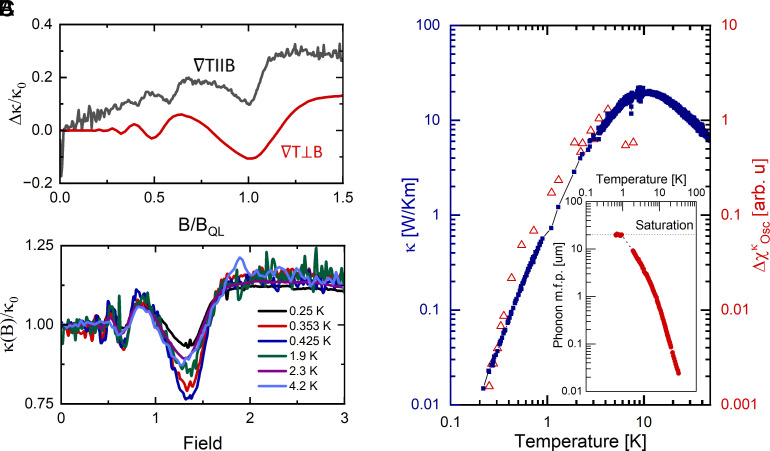
Temperature dependence of the magneto-thermal quantum oscillation amplitude in ZrTe_5_. (*A*) Comparison of transverse (red) and longitudinal (black) magneto-thermal conductivity. For fields applied along the thermal gradient direction, a clear linear contribution to conductivity is seen. The magnetic field strength is normalized to $B_{\mathrm{QL}}$, the magnetic field strength above which only the lowest Landau level is occupied. (*B*) Temperature dependence of transverse magneto-thermal conductivity divided by $\kappa_{0}$. (*C*) Comparison of zero field thermal conductivity (blue) with the temperature dependence of the quantum oscillation amplitude. Both quantities follow a similar temperature dependence suggesting a common origin; *Inset* shows the temperature dependence of the phonon mean free path.

Although quantum oscillations are commonly attributed to electrons and the existence of a Fermi surface, the dominance of the lattice degrees of freedom in thermal transport at zero field suggests that the huge variations of the thermal conductivity in magnetic fields originate from changes of the phonon’s mean free path due to the scattering on the electrons. To confirm the origin of the giant magneto-thermal quantum oscillations in ZrTe_5_, we measured the temperature dependence of the magneto-thermal quantum oscillations down to 250 mK. Comparison of the temperature dependence of the amplitude of the last oscillation with the zero-field thermal conductivity reveals that both quantities follow approximately a $T^{3}$ dependence, Fig. 3*C*, strongly suggesting that here, the quantum oscillations seen in thermal transport indeed are rooted in the phononic degrees of freedom. Indeed plotting κ(B) divided by κ(B=0) reveals good collapse of the data, Fig. 3*B*.

Recently, similar giant quantum oscillations have been reported in the longitudinal thermal conductivity (∇T‖B) of the Weyl semimetals TaAs and NbP (10, 11). In these cases, the oscillations were attributed to the presence of CZS—a collective bosonic excitation of the Fermi sea of chiral fermions that requires multiple Fermi pockets. An alternative explanation was put forward for the presence of giant oscillations in NbP in a transverse-field configuration (∇T⊥B): The presence of both electrons and holes was suggested to reduce the electrical conductivity without affecting the thermal conductivity, giving rise to a violation of the Wiedemann–Franz. Although both explanations seem feasible in the case of Weyl fermion materials harboring both electron and hole-like Fermi pockets, this is not the case in ZrTe_5_. The samples studied in the present work have been shown (13, 16) to contain only a single electron-like Fermi surface with a massive Dirac dispersion. In addition with the Fermi level of ∼40 to 100 K and the Dirac mass-gap of the order of 100 K, an influence of thermally excited holes on thermal transport is excluded at the relevant temperatures, precluding the influence of both effects on thermal transport in the studied samples.

We now argue that a thermal current carried dominantly by phonons can display quantum oscillations originating from giant oscillations of the absorption rate of phonons by the electrons. Moreover, we show that such oscillations appear both when the thermal current is directed parallel but also perpendicular to the magnetic field B. Since a detailed theory of the phonons’ thermal conductivity would be material specific, we resort to a general argument based on a phase-space argument to unveil the origin of these quantum oscillations. We argue there is a strong enhancement of the absorption of the thermal phonons by the electrons for specific values of the magnetic field. The phonon contribution to the thermal conductivity can be expressed as (2)

[1] $$\kappa =\frac{1}{3}C_{p}{\bar{v}}_{\text{s}}l_{\text{ph}},$$

where ${\overset{¯}{v}}_{s}$ is the averaged phonon’s velocity, the specific heat scales as $C_{p}\propto T^{3}$ at low temperatures, while at B=0, the phonon mean free path $l_{\text{ph}}≃20$ μm is temperature independent; see *Inset* of Fig. 3*C*. As the magnetic field is increased, successive minima of the conductivity κ(B), Fig. 3*A*, manifest a large decrease of this mean free path, signaling an enhanced absorption rate of phonons. By expressing $l_{\text{ph}}={\bar{v}}_{\text{s}}\tau_{\text{ph}}$, we can sum the different contributions to the phonon scattering rate $\tau_{\text{ph}}^{-1}$ by resorting to the Matthiessen’s rule. At low temperatures, the phonon–phonon Umklapp scattering is greatly reduced (30). The conduction electrons themselves constitute the main mechanism for scattering phonons, besides the sample surface and grain boundary scattering which is magnetic field independent.

**Fig. 4.**
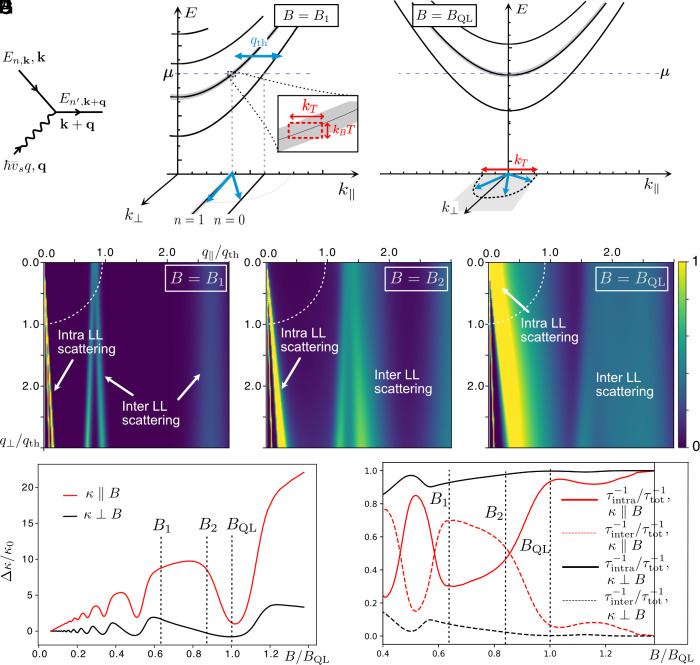
Quantum oscillations of thermal conductivity via phonon absorption by electrons. (*A*) Energy and momentum conservation constrain the absorption of a phonon of momentum q by an electron between two LLs $E_{n,k}$ and $E_{n^{\prime },k+q}$. (*B*) When the chemical potential lies away from the *Bottom* of Landau levels, only a subset of phonon’s momenta can be absorbed by electrons in inter-LL transitions and the phonon’s absorption is very anisotropic. The typical thermal phonon’s momenta $q_{\text{th}}=k_{\text{B}}T/{\bar{v}}_{\text{s}}$ for allowed processes are graphically represented. (*C*) When the chemical potential crosses the *Bottom* of a Landau level n, the absorption of phonons in intra-LL transitions within the level n becomes efficient and almost isotropic. (*D*–*F*) The momentum dependence of the phonon’s absorption rate ħ/τ(q) is represented for different magnetic fields, for T=2 K, with the colors encoding its intensity. (*D* and *E*) Away from the *Bottom* of a Landau level, for $B=B_{1},B_{2}$, inter-LL processes dominate the phonon absorption in the direction of B. They absorb inefficiently phonons of momenta $q≳q_{\text{th}}$. (*F*) At the *Bottom* of a Landau level for $B=B_{\text{QL}}$, the absorption becomes dominated by efficient inter-LL transitions, manifested by a sudden increase of the absorption rate and a minimum of the thermal conductivity. (*G*) The resulting relative thermal conductivity $\Delta \kappa \left(B\right)/\kappa_{0}=\tau \left(B\right)/\tau_{0}$ is plotted at T=2 K, where $\kappa_{0}=\kappa \left(B=0\right)$, in the direction parallel and perpendicular to B. (*H*) The inter- and intra-LL transition’s rates are plotted relative to the total absorption rate $\tau_{\text{tot}}^{-1}=\tau_{\text{inter}}^{-1}+\tau_{\text{intra}}^{-1}$ as a function of the rescaled magnetic field $B/B_{\text{QL}}$, for a temperature T=2 K. In the direction of B the inter-LL absorption mechanism dominates the absorption rate away from the quantum oscillations, giving rise to the κ∝B scaling in panel (*E*). In contrast, perpendicular to the magnetic field, intra-LL processes dominate the phonon’s absorption irrespective of the magnetic field, leading to an constant average κ.

In the presence of a strong magnetic field B, the dynamics of the electrons transverse to B freezes, leading to one-dimensional dispersive LLs $E_{n,k_{\|}}$, represented in Fig. 4*B*, indexed by n notand the electron’s momentum parallel to the field $k_{\|}$, here chosen along the b crystallographic axis:

[2a] $$\begin{array}{cc} E_{0,k_{\|}} & =\pm \sqrt{{\left(v_{b}\hbar k_{\|}\right)}^{2}+m^{2}}\;, \end{array}$$

[2b] $$\begin{array}{cc} E_{n,k_{\|}} & =\pm \sqrt{{\left(v_{b}\hbar k_{\|}\right)}^{2}+m^{2}+2ne\left|B\right|\hbar v_{a}v_{c}}\;. \end{array}$$

with $v_{a,b,c}$ the Fermi velocity along the a,b,c crystallographic axis and m a small mass parameter m=10meV. We neglected a subdominant Zeeman energy for the sake of clarity. Each of these levels has a density of states ρ(B)=eB/(2πħ) associated to the different momenta $k_{⊥}$. Thermal phonons have a typical energy $\hbar \omega ≲k_{\text{B}}T$ and momentum $\left|q\right|≲q_{\text{th}}=k_{B}T/\left(\hbar {\bar{v}}_{\text{s}}\right)$. The absorption and emission of these phonons by the electrons is constrained by energy and momentum conservation which depend on the Landau levels; see Fig. 4*A*:

[3] $$k^{\left(\text{in}\right)}+q=k^{\left(\text{out}\right)}\;;\;E_{n,k_{\|}^{\left(\text{in}\right)}}+\hbar \omega =E_{n^{\prime },k_{\|}^{\left(\text{out}\right)}}\;.$$

In Fig. 4 *B* and *C*, we represent schematically these conditions. From Fermi’s golden rule, the typical phonon’s scattering rate $\tau_{\text{ph-e}}^{-1}$ can be expressed as

[4] $$\tau_{\text{ph-e}}^{-1}=\int_{q}\;\tau^{-1}\left(q\right)/\left(\exp\left(\beta \hbar {\bar{v}}_{\text{s}}q\right)-1\right),$$

with $\beta^{-1}=k_{\text{B}}T$ and a scattering rate for phonons of momentum q that follows the energy and momentum conservation rules:

[5] $$\begin{array}{cc} \hbar \tau^{-1}(q)=\underset{n,n^{\prime }}{\sum } & \int_{k}f\left(E_{n,k}\right)\left(1-f(E_{n^{\prime },k+q})\right)\\ & \delta \left(E_{n^{\prime },k+q}-E_{n,k}-\hbar {\bar{v}}_{\text{s}}|q|\right){\left|V_{k+q;k,q}\right|}^{2}, \end{array}$$

with f(E) the Fermi–Dirac distribution function. In a phase-space argument, we evaluate the variation of the scattering rate (Eq. **5**) by neglecting the variation of the matrix element of the electron–phonon coupling potential $V_{k+q;k,q}$, focusing on the energy and momentum constraints. The dependence on the phonon momentum q of this scattering rate is represented in Fig. 4*D*–*F* for different values of the magnetic field B; see *Numerics* for details on the numerical method.

The strong anisotropy of the Landau levels is reflected in how different mechanisms dominate the absorption in various directions of phonon propagation. Along the magnetic field, only a few discrete momenta $q_{\|}$ can be absorbed via inter-LL transitions away from the bottom of a LL; see Fig. 4 *B* and *D*. This results in a rather inefficient absorption at high magnetic fields and a peak in the thermal conductivity; see Fig. 4*G*. The absorption rate along the field is thus set by the number of relevant inter-LL transitions. The number of LLs (Eq. **2**) crossing the chemical potential scales as ∝1/B, leading to a number of inter-LL transitions scaling as $1/B^{2}$. Hence, accounting for the electronic density of LLs, the resulting average absorption rate (Eq. **5**) scale as $\hbar {\overset{¯}{\tau }}^{-1}\propto 1/B$, as shown on the net linear increase in field of κ‖B shown in Fig. 4*G*. In the direction perpendicular to B, the absorption is dominated by intra-LL transitions, see Fig. 4 *D* and *E*, and it is very directional, as shown in Fig. 4*H*. As a consequence, the absorption rate perpendicular to the field is almost constant for fields away from the bottom of LLs, leading to an average constant κ⊥B as opposed to the linear increase of κ‖B shown in Fig. 4*G*.

At the bottom of each Landau band, where $\mu ≃E_{n,k=0}$, the absorption mechanism becomes dominated by intra-LL transitions within the level n both along and perpendicular to B. Simultaneously, absorption of small phonon momenta in all directions becomes possible, leading to a sudden increase of the efficiency of this absorption, as shown in Fig. 4*F*. This sudden increase of the absorption rate $\hbar /\tau_{\text{tot}}$ for each LL leads to the observed quantum oscillations of the phonon-dominated thermal conductivity, as shown in the theoretical curves of Fig. 4*G*.

## Discussion

We have shown that thermal conductivity dominated by phonons can display giant quantum oscillations due to the increased phonon absorption by electrons at the bottom of Landau levels. One could argue that presence of such an effect could be corroborated by direct measurements of the sound attenuation. Indeed, while its relation to thermal conductivity was not discussed, oscillations in the sound absorption in relation to Landau levels for magnetic fields parallel to the sound propagation direction were discussed as early as the 1960s (31, 32); see also section 12.8 of ref. 2.

To contrast the experimental manifestation of quantum oscillations in both quantities, we have conducted sound attenuation measurements in our samples, Fig. 2 *C* and *F*. They reveal that the relative changes in the attenuation and the speed of sound due to magneto-acoustic quantum oscillations do not exceed 1%, This appears at odds with the proposed attenuation mechanism. Indeed, recently it has been argued (11) that since attenuation measurements probe only the phonon degrees of freedom, it is expected that strong quantum oscillations appearing in thermal conductivity due to phonon attenuation by electrons should manifest as a strong variation of the echo amplitude.

This apparent contradiction is resolved by realizing that thermal-conductivity and sound-attenuation measurements probe very different energy scales. In ultrasonic measurements, one typically probes the phonons with frequencies ≲1 GHz, whereas thermal conductivity probes thermal phonons whose frequencies, even at 200 mK, exceed 3 GHz. Attenuation of phonons by electrons can be separated into two regimes: The low-frequency “hydrodynamic” regime where the phonon wavelength is much larger than the electron mean free $q\cdot l_{m.f.p}\ll 1$ path and attenuation increases quadratically with phonon frequency and the other regime is the “quantum limit” of attenuation $q\cdot l_{m.f.p}\gg 1$, where attenuation increases linearly with frequency (33). In the case of ZrTe_5_, the sound wavelength at 314 MHz is ∼10 μm, and the electronic mean free path at ≲2 K is of the order of 1 μm placing the ultrasound measurement in the hydrodynamic limit. In contrast, typical phonons probed in thermal conductivity at 200 mK have a wavelength of ∼0.1 μm, well in the quantum limit of absorption. This makes phonon absorption at the frequencies probed by thermal conductivity measurements more than two orders of magnitude stronger than in typical ultrasound experiments, making it a much more sensitive technique to study electron–phonon processes (34). Thermal conductivity appears as an ideal probe of the giant quantum oscillations of the phonon absorption rate by electrons.

Although, for clarity, we have focused our experiments and modeling on one of the simplest Dirac semimetals, ZrTe_5_, there is ample experimental evidence suggesting the generality of the proposed mechanism in semimetals whose thermal transport is dominated by phonons. Signatures of quantum oscillations in κ, tentatively attributed to phonon absorption, have been seen in Bi (35) at magnetic fields where only a few LL were occupied. Similarly, quantum fluctuations of the phonon-dominated thermal conductivity of Sb were attributed to fluctuations in the number of scattering centers for phonons in ref. 36. In addition, here the longitudinal thermal conductivity displayed a linear in B behavior besides quantum fluctuations, characteristic of the inter-LL absorption processes described in the present paper. More recently, studies on Sb (37) have also found a regime where the thermal conductivity was dominated by phonons, yet displayed quantum oscillations of their thermal conductivity; this was attributed to a momentum exchange between phonons and electrons. In addition, weak quantum oscillations and a linear-in-B dependence were also observed in the thermal conductivity of graphite (38) and graphene (39).

Recently thermal-conductivity measurements have taken a central stage in the study of quantum matter due to their potential to reveal exotic low-energy excitation’s such as charge neutral modes (see e.g. refs. 40–43).Traditionally, in thermal transport measurements, one seeks additional nontrivial contributions to thermal conductivity on top of a simple phononic background. Surprisingly, recent studies of the thermal Hall effect have shown that scattering of phonons from electronic or spin subsystem can lead emergence of nontrivial contributions in the phononic thermal Hall conductivity (44–48). Here, we show that the more traditional longitudinal phononic thermal transport can also acquire a nontrivial field dependence due to scattering from electronic excitations. The proposed phase-space mechanism is general and should be applicable to any thermal measurement in magnetic fields, including the measurements needed to interpret more exotic phenomena like the Thermal Hall.

## Materials and Methods

### Sample Synthesis and Preparation.

High-quality single-crystalline ZrTe_5_ samples were synthesized using high purity elements (99.9999% zirconium and 99.9999% tellurium). Needle-shaped crystals (about 0.2×0.3×3 mm^3^) were obtained by a tellurium flux method. Prior to transport measurements, Pt contacts were sputter deposited on the sample surface to ensure low contact resistance. The contact geometry was defined using Al hard masks. This procedure allowed us to achieve contact resistances below 1 Ω. Band-structure parameters of the main sample have been studied in ref. 13, where it is refereed to as sample B.

### Sample Environment.

All transport measurements up to 9 T were performed in a temperature-variable cryostat (PPMS Dynacool, Quantum Design), equipped with a dilution refrigerator insert. Additional longitudinal thermal transport experiments were performed in a 22 T Oxford instruments cryostat equipped with a ^3^He insert. In addition ultrasound measurements were performed in a 16 T Oxford instruments cryostat equipped with a standard Variable Temperature Insert (VTI) and Kelvinox MX400 Dilution refrigerators.

### Ultrasound experiments.

Ultrasound measurements were performed using a phase-sensitive pulse-echo technique. Two piezoelectric lithium-niobate (LiNbO_3_) resonance transducers were glued to opposite parallel surfaces of the sample to excite and detect acoustic waves. The sample surfaces were polished using a focused Ion beam in order to ensure that the transducer attachment surfaces were smooth and parallel. The longitudinal acoustic waves were propagated along the a-axis. Relative sound-velocity Δv/v, and sound attenuation Δα, were measured for field applied along the a. Data showing propagation with field along the b-axis have been previously published in ref. 13.

### Periodicity of the Observed Oscillations.

**Fig. 5.**
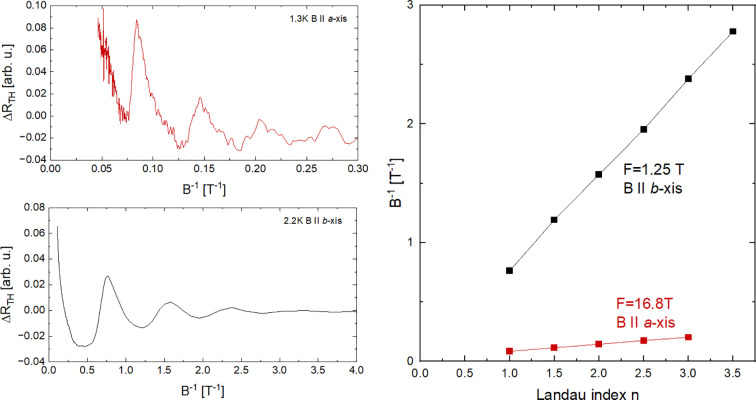
*Left* panel: Thermal resistance for both field orientation plotted as in 1/B, *Right* panel, fan diagram of both datasets highlighting periodicity of the oscillations in 1/B).

In order to check weather oscillations seen in thermal transport are periodic we have extracted the oscillating part of thermal conductivity by subtracting a linear background and plotted the resulting curves in 1/B, see Fig. 5. The oscillations appear perfectly periodic in inverse field. This is additionally confirmed by constructing Landau fan diagrams clearly showing that indexes of both maxima and minima seen in thermal resistance are linearly related to 1/B. In particular, the extracted slopes are in excellent agreement with frequencies extracted in previous studies (13).

### Numerics.

For the numerical evaluation of the phonon-scattering rates for phonons of momentum q, associated to a transfer of an electron from Landau level n to Landau level n′ (Eq. **5**), we first perform the integration on the variable $k_{⊥}$, leading to a density of states per Landau level equal to $\frac{e|B|}{2\pi \hbar }$. Then, to integrate over $k_{\|}$, we determined the zeros of

[6] $$f_{q}\left(k_{\|}\right)=E_{n\prime ,k_{\|}+q_{\|}}-E_{n,k_{\|}}-\hbar {\bar{v}}_{\text{s}}\left|q\right|$$

by using a dichotomy algorithm. The integral can then be evaluated by using the property of the δ(x) distribution, for a function f(x) vanishing at positions $x\in \left\{x_{0},x_{1},...,x_{n}\right\}$

[7] $$\delta \left(f\left(x\right)\right)=\sum_{k=0}^{n}\frac{\delta (x-x_{k})}{\left|{\left.\partial_{x}f\right|}_{x=x_{k}}\right|}\;.$$

After summation on n and n′, the result of these integrations at a T=2K is plotted in Fig. 4*D*–*F* for three different values of magnetic-field.

Afterward, it is possible to determine the total scattering rate by evaluating the integral over the phonon momentum q, taking into account the thermal occupation of the phonon states captured by a Bose–Einstein function.

The experiment measured longitudinal and transverse transport. To capture the difference in between these two situations numerically, we defined $\tau_{⊥}$ and $\tau_{\|}$, such that $\kappa^{\|/⊥}=\frac{1}{3}C_{p}{\overset{¯}{v}}_{\text{s}}^{2}\tau_{\|/⊥}$, and evaluated them by assuming that the phonons participating in the transverse and longitudinal transport measurement are those contained in a solid angle $\frac{\pi }{6}$ around the transport direction, such for example that

[8] $$\tau_{\text{ph-e,}\|}^{-1}=\int_{q}\;\frac{\tau^{-1}\left(q\right)}{\exp(\beta \hbar {\bar{v}}_{\text{s}}q)-1}\theta \left(q_{\|}-2q_{⊥}\right)\;,$$

with θ(x) the Heaviside function.

## Supplementary Material

Appendix 01 (PDF)

## Data Availability

All data needed to evaluate the conclusions in the paper are presented in the paper and/or *SI Appendix*.

## References

[1] N. W. Ashcroft, N. D. Mermin, Solid State Physics (Cengage Learning, 2022).

[2] A. A. Abrikosov, Fundamentals of the Theory of Metals (Courier Dover Publications, 2017).

[3] L. Schubnikow, W. J. De Haas, Magnetische widerstandsvergrösserung in einkristallen von wismut bei tiefen temperaturen. Proc. R. Neth. Acad. Arts Sci. **33**, 130–133 (1930).

[4] L. Schubnikow, W. J. De Haas, Neue erscheinungen bei der widerstandsänderung von wismuthkristallen im magnetfeld bei der temperatur von flüssigem wasserstoff (i). Proc. R. Neth. Acad. Arts Sci. **33**, 363–378 (1930).

[5] I. Lifshitz, L. Kosevich, On the theory of the shubnikov-de haas effect. Sov. Phys. JETP **6**, 67–77 (1958).

[6] W. De Haas, P. Van Alphen, The dependence of the susceptibility of diamagnetic metals upon the field. Proc. Neth. R. Acad. Sci. **33**, 170 (1930).

[7] S. Shalyt, The thermal conductivity of bismuth at low temperatures. J. Phys. (USSR) **8**, 315–316 (1944).

[8] W. Pratt Jr., C. Uher, Thermal conductivity of bismuth at ultralow temperatures. Phys. Lett. A **68**, 74–76 (1978).

[9] R. Berman, Thermal Conduction in Solids (Oxford University Press, 1976).

[10] P. K. Tanwar, M. S. Alam, M. Ahmad, D. Kaczorowski, M. Matusiak, Severe violation of the Wiedemann-Franz law in quantum oscillations of NbP. Phys. Rev. B **106**, L041106 (2022).

[11] J. Xiang , Giant magnetic quantum oscillations in the thermal conductivity of TaAs: Indications of chiral zero sound. Phys. Rev. X **9**, 031036 (2019).

[12] U. Stockert , Thermopower and thermal conductivity in the Weyl semimetal NbP. J. Phys. Condens. Matter **29**, 325701 (2017). 28628029 10.1088/1361-648X/aa7a3b

[13] S. Galeski , Origin of the quasi-quantized hall effect in ZrTe_5_. Nat. Commun. **12**, 3197 (2021). 34045452 10.1038/s41467-021-23435-y PMC8159947

[14] M. Hasan, S. Y. Xu, I. Belopolski, S. M. Huang, Discovery of Weyl fermion semimetals and topological fermi arc states. Annu. Rev. Condens. Matter Phys. **8**, 289–309 (2017).

[15] F. Tang , Three-dimensional quantum hall effect and metal-insulator transition in ZrTe_5_. Nature **569**, 537–541 (2019). 31068693 10.1038/s41586-019-1180-9

[16] S. Galeski , Signatures of a magnetic-field-induced lifshitz transition in the ultra-quantum limit of the topological semimetal ZrTe_5_. Nat. Commun. **13**, 7418 (2022). 36456570 10.1038/s41467-022-35106-7 PMC9715529

[17] Y. Zhang , Electronic evidence of temperature-induced lifshitz transition and topological nature in ZrTe_5_. Nat. Commun. **8**, 15512 (2017). 28534501 10.1038/ncomms15512 PMC5457516

[18] P. Zhang , Observation and control of the weak topological insulator state in ZrTe_5_. Nat. Commun. **12**, 406 (2021). 33462222 10.1038/s41467-020-20564-8 PMC7813838

[19] P. Wang , Approaching three-dimensional quantum hall effect in bulk HfTe_5_. Phys. Rev. B **101**, 161201(R) (2020).

[20] C. Wang, Thermodynamically induced transport anomaly in dilute metals ZrTe_5_ and HfTe_5_. Phys. Rev. B **126**, 126601 (2021). 10.1103/PhysRevLett.126.126601 33834821

[21] M. Hooda, C. Yadav, Enhanced thermopower and low thermal conductivity in p-type polycrystalline ZrTe_5_. Appl. Phys. Lett. **111**, 053902 (2017).

[22] C.-W. Cho , Thermal transport properties and some hydrodynamic-like behavior in three-dimensional topological semimetal ZrTe_5_. Phys. Rev. B **085132**, 105 (2022).

[23] M. T. Terry, Ed., Semiconductors and Semimetals (Elsevier, 2001), vol. 70, pp. 179–206.

[24] B. M. Zawilski, R. T. Littleton, T. M. Tritt, Investigation of the thermal conductivity of the mixed pentatellurides. Appl. Phys. Lett. **77**, 2319–2321 (2000).

[25] P. Behera, M. M. Patidar, U. P. Deshpande, R. Venkatesh, V. Ganesan, Transport and thermal properties of polycrystalline ZrTe_5_. J. Appl. Phys. **127**, 235110 (2020).

[26] R. Shaviv, E. F. Westrum, H. Fjellvåg, A. Kjekshus, ZrTe_5_ and HfTe_5_: The heat capacity and derived thermophysical properties from 6 to 350 k. J. Solid State Chem. **81**, 103–111 (1989).

[27] F. Arnold , Negative magnetoresistance without well-defined chirality in the Weyl semimetal tap. Nat. Commun. **7**, 11615 (2016). 27186980 10.1038/ncomms11615 PMC4873626

[28] A. Pippard, Magnetoresistance in Metals (Cambridge University Press, 1989).

[29] R. D. dos Reis, On the search for the chiral anomaly in Weyl semimetals: The negative longitudinal magnetoresistance. New J. Phys. **18**, 085006 (2016).

[30] J. M. Ziman, Electrons and Phonons: The Theory of Transport Phenomena in Solids (Oxford University Press, 2001).

[31] V. Gurevich, V. Skobov, Y. A. Firsov, Giant quantum oscillations in the acoustical absorption by a metal in a magnetic field. Sov. Phys. JETP **13**, 552 (1961).

[32] Y. Shapira, B. Lax, Line shape and amplitude of giant quantum oscillations in ultrasonic absorption. Phys. Rev. **138**, A1191 (1965).

[33] B. Lüthi, Physical Acoustics in the Solid State (Springer Science & Business Media, 2007), vol. 148.

[34] M. Holland, Thermal conductivity and ultrasonic attenuation. IEEE Trans. Ultrason. **15**, 18–27 (1968).

[35] M. Steele, J. Babiskin, Oscillatory thermomagnetic properties of a bismuth single crystal at liquid helium temperatures. Phys. Rev. **98**, 359 (1955).

[36] J. R. Long, C. Grenier, J. Reynolds, Electron and lattice transport phenomena in an antimony crystal at liquid-he 4 temperatures. Phys. Rev. **140**, A187 (1965).

[37] A. Jaoui , Formation of an electron-phonon bifluid in bulk antimony. Phys. Rev. X **12**, 031023 (2022).

[38] C. Ayache, M. Locatelli, “Quantum oscillations effect on the lattice thermal conductivity of graphite under very high magnetic fields” in Phonon Scattering in Condensed Matter, H. Maris, Eds. (Springer, 1980), pp. 465–467.

[39] J. D. Crossno, “Electronic thermal conductance of graphene via electrical noise,” PhD thesis, Harvard University (2017).

[40] N. Wakeham , Gross violation of the Wiedemann-Franz law in a quasi-one-dimensional conductor. Nat. Commun. **2**, 396 (2011). 21772267 10.1038/ncomms1406 PMC3144592

[41] M. Sato, S. Fujimoto, Majorana fermions and topology in superconductors. J. Phys. Soc. Jpn. **85**, 072001 (2016).

[42] Y. Kasahara , Majorana quantization and half-integer thermal quantum hall effect in a Kitaev spin liquid. Nature **559**, 227–231 (2019). 10.1038/s41586-018-0274-0 29995863

[43] Y. Sato , Unconventional thermal metallic state of charge-neutral fermions in an insulator. Nat. Phys. **15**, 954–959 (2019).

[44] L. Mangeolle, L. Balents, L. Savary, Phonon thermal hall conductivity from scattering with collective fluctuations. Phys. Rev. X **12**, 041031 (2022).

[45] B. Li, B. Fauqué, Z. Zhu, K. Behnia, Phonon thermal hall effect in strontium Titanate. Phys. Rev. Lett. **124**, 105901 (2020). 32216396 10.1103/PhysRevLett.124.105901

[46] X. Li , The phonon thermal hall angle in black phosphorus. Nat. Commun. **14**, 1027 (2023). 36823192 10.1038/s41467-023-36750-3 PMC9950068

[47] H. Guo, D. G. Joshi, S. Sachdev, Resonant thermal hall effect of phonons coupled to dynamical defects. Proc. Natl. Acad. Sci. U.S.A. **119**, e2215141119 (2022). 36367907 10.1073/pnas.2215141119 PMC9674268

[48] X. Li , Large phonon thermal hall conductivity in the antiferromagnetic insulator Cu_3_TeO_6_. Proc. Natl. Acad. Sci. U.S.A. **119**, e2208016119 (2022). 35969770 10.1073/pnas.2208016119 PMC9407214

