# Supplementary material for: Giant quantum oscillations in thermal transport in low-density metals via electron absorption of phonons
Source: Proc Natl Acad Sci U S A. 2025 Mar 5;122(10):e2408546122. doi: 10.1073/pnas.2408546122 (PMC11912444; doi:10.1073/pnas.2408546122)
Supplement: Supplementary file 1 — Appendix 01 (PDF) [file pnas.2408546122.sapp.pdf]

Supplementary information

B. Bermond,[1,*] R. Wawrzyńczak,[2] S. Zherlitsyn,[3] T. Kotte,[3] T. Helm,[3] D. Gorbunov,[3] G. D. Gu,[4] Q. Li,[4] F. Janasz,[5] T. Meng,[6] F. Menges,[2] C. Felser,[2] J. Wosnitza,[3,7] Adolfo G. Grushin,[8,†] David Carpentier,[1,‡] J. Gooth,[2,9] and S. Galeski[9,2,3,§]

[1] *ENSL, CNRS, Laboratoire de Physique, F-69342 Lyon, France*

[2] *Max Planck Institute for Chemical Physics of Solids, Nöthnitzer Straße 40, 01187 Dresden, Germany*

[3] *Hochfeld-Magnetlabor Dresden (HLD-EMFL) and Würzburg-Dresden Cluster of Excellence ct.qmat, Helmholtz-Zentrum Dresden-Rossendorf, 01328 Dresden, Germany*

[4] *Condensed Matter Physics and Materials Science Department, Brookhaven National Laboratory, Upton, NY, USA*

[5] *Department of Engineering, Faculty of Science, Medicine and Technology, University of Luxembourg, Luxembourg*

[6] *Institute for Theoretical Physics and Würzburg-Dresden Cluster of Excellence ct.qmat, Technische Universität Dresden, Dresden, Germany*

[7] *Institut für Festkörper-und Materialphysik and Würzburg-Dresden Cluster of Excellence ct.qmat, Technische Universität Dresden, 01062 Dresden, Germany*

[8] *Univ. Grenoble Alpes, CNRS, Grenoble INP, Institut Néel, 38000 Grenoble, France*

[9] *Physikalisches Institut, Universität Bonn, Nussallee 12, 53115 Bonn, Germany*

I. ADDITIONAL DATA

A. Field dependence of specific heat of $ZrTe_5$ rystals.

In our analysis, we have assumed that the specific heat of $ZrTe_5$ is approximately independent of magnetic field and, thus, the variations of the phonon thermal conductivity $\kappa = \frac{1}{3} \cdot C_\mathrm{p} \cdot \overline{v}_\mathrm{s} \cdot l_\mathrm{ph}$ in magnetic field are given by changes of l_ph. To verify if this assumption holds we have measured the specific heat of $ZrTe_5$ at 2 K using a standard PPMS specific heat option, using a field calibrated puck. The results shown in Fig.1 confirm our assumption. Field-induced variations of $C_\mathrm{p}(H)$ do not exceed 1% and, thus cannot account for the almost 20% changes of κ. In particular, $C_\mathrm{p}(H)$ does not exhibit any signs of quantum oscillations.

* baptiste.bermond@ens-lyon.fr
† adolfo.grushin@neel.cnrs.fr
‡ david.carpentier@ens-lyon.fr
§ sgaleski@uni-bonn.de

Material	Density	Thermal conductivity	Heat capacity
	g/cm3	*W/mK*	*J/kgK*
Manganin	8.4	0.0335	0.8
Sapphire	3.98	2.4	0.02
Silicon	2.3	200	0.005
Silver	10.49	3940	1
Steel	7.85	60.5	434
ZrTe5	5.6	0.7	0.005

Table I. Material properties used in ANSYS simulations

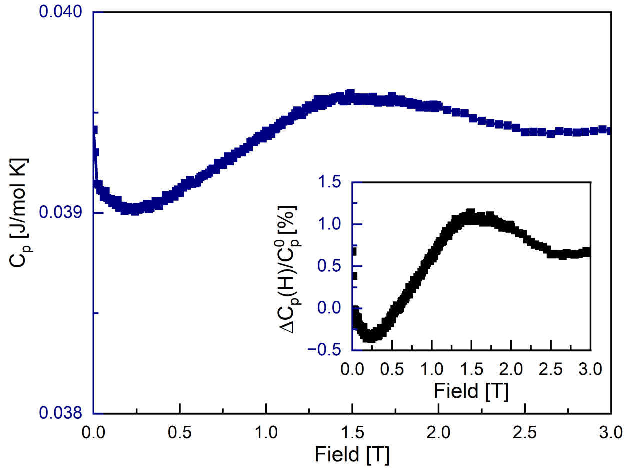

Figure 1. Field ($B \parallel b$-axis) dependance of the specific heat measured at 1.9 K. The inset shows the change of C_p in percent. The field induced changes amount to maximum 1% of the total specific heat and, thus, cannot account for the huge quantum oscillations seen in thermal transport.)

B. Signatures of current jetting in longitudinal charge transport measurements.

Current jetting is a phenomenon, where the electrical current distribution within a sample is highly non-uniform in measurements of the longitudinal magneto-resistance. In particular, in samples with low charge carrier density current does not "even out" after injection through imperfect contacts and can form "jets" of current along the direction of the magnetic field. This uneven distribution of current can lead to misleading results in experiments such as appearance of negative magnetoresistance [1–3]. One method for determining weather current jetting is present in resistance measurements is by applying current to a pair of contacts located on one side of the sample and comparing voltage drops measured on the same and opposite sides of the sample [2] as, see sketch of the measurement geometry in Fig. 2. Results of such a measurement performed on $ZrTe_5$ samples at 2 K are shown in Fig. 2. Measurements of the voltage drop on opposite sides of the sample display a dramatically different field dependence strongly suggesting presence of current jetting and making longitudinal magneto-resistance measurements unreliable. Current jetting is not relevant in measurement of the thermal conductivity since there are no current injection point in such measurement.

II. ESTIMATING THE TEMPERATURE DISTRIBUTION ACROSS THE THERMAL TRANSPORT SETUP

In this work we have used a custom build thermal transport setup. In order to estimate heat flow direction and estimate potential heat losses to gauge whether the sample is appropriately isolated form the outside world we have simulated the temperature distribution across the setup using a finite elements thermal transport solver included in the ANSYS package. In the computation we have included radiation effects and used material parameters tabulated in Table 1 reflecting the materials used in design of the experiment.

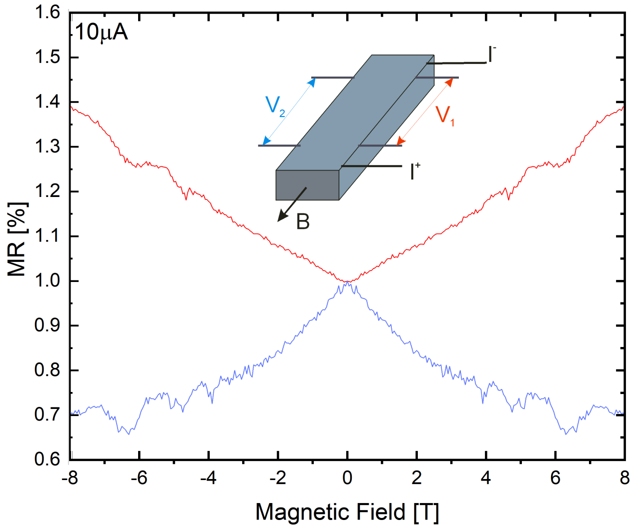

Figure 2. Signatures of current jetting in longitudinal charge transport measurements in $ZrTe_5$. The magnetic field and electric current are both applied along the a-axis. In this experiment current was applied via contacts located on the left side of the crystal. Radically different field-dependant voltage drops mat the contacts V_1 and V_2 evidence that the current distribution in this measurement configuration is not homogeneous.)

**Heating BC**	Heat generation rate	Equivalent power
	W/m3	*uW*
Simulation 1	0.116e4	0.025
Simulation 2	4.17e4	0.9
Simulation 3	11.6e4	2.5
**Radiation BC**	Emissivity	Ambient temperature
		K
In all simulations	0.4	2
**Temperature BC**	Temperature at contacts	
	K	
In all simulations	1	

Table II. Applied boundary conditions

Thermal conductance of the manganin wires has been adjusted to reflect thermal conductance of the real length of used wire used in the experiment. The calculation domain shown in Fig.3 is composed of the ZrTe5 sample, two thermistors and a heater, connected with silver and manganing wires respectively - reflecting the used experimental setup. Constant temperature boundary condition was applied to the end of the sample, representing a connection to the heat bath and to end of each wire. In addition, radiative boundary condition was applied to the ZrTe5 sample to simulate the heat losses. Finally, volumetric heat generation was included in the heater element, to simulate the Joule heating used in the experiment. Table 2 shows a summary of used values.

Simulations results shown on Fig.4 and Fig.5 confirm that in our design most heat flow occurs from the heater to the thermal bath via ZrTe5 sample and thus our measured sample thermal conductivity's are expected to closely reflect the actual values of thermal conductivity. Table 3 summarizes the heat flow in ZrTe5 for all three used heating powers.

Investigation of Fig.4 and Fig.5 confirms that in our design most heat generated by the heater is transported through the sample to the thermal bath and thus our measured sample thermal conductivity's are expected to closely reflect the actual values of thermal conductivity.

Heatflow into ZrTe5	Heatflow via ZrTe5 to thermal bath	Difference
uW	uW	%
0.025	0.0246	1.65
0.9	0.0885	1.65
2.5	2.46	1.65

Table III. Heat flow comparison for ZrTe5 sample in simulations

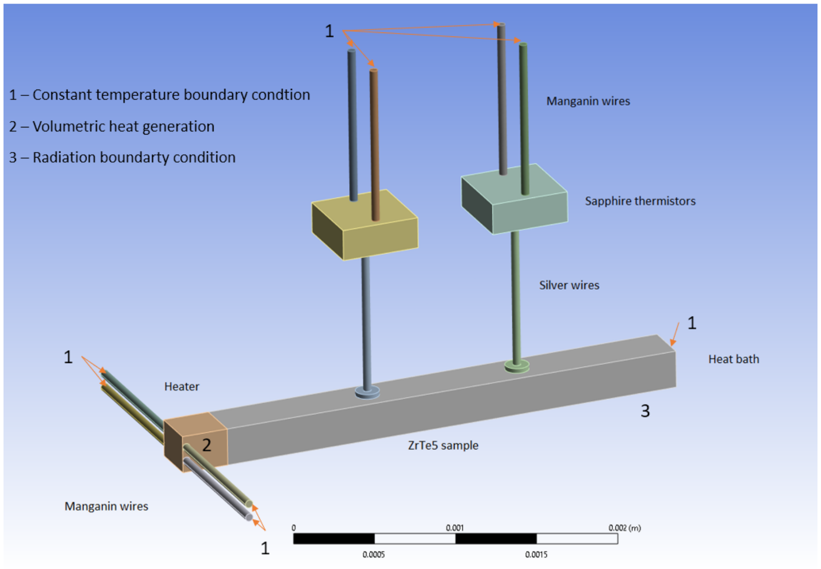

Figure 3. Thermal boundary conditions applied

[1] A. Pippard, *Magnetoresistance in Metals* (Cambridge University Press, 1989).
[2] F. Arnold, C. Shekhar, S. Wu, *et al.*, Nature Communications **7**, 11615 (2016).
[3] R. D. d. Reis *et al.*, New J. Phys. **18**, 085006 (2016).

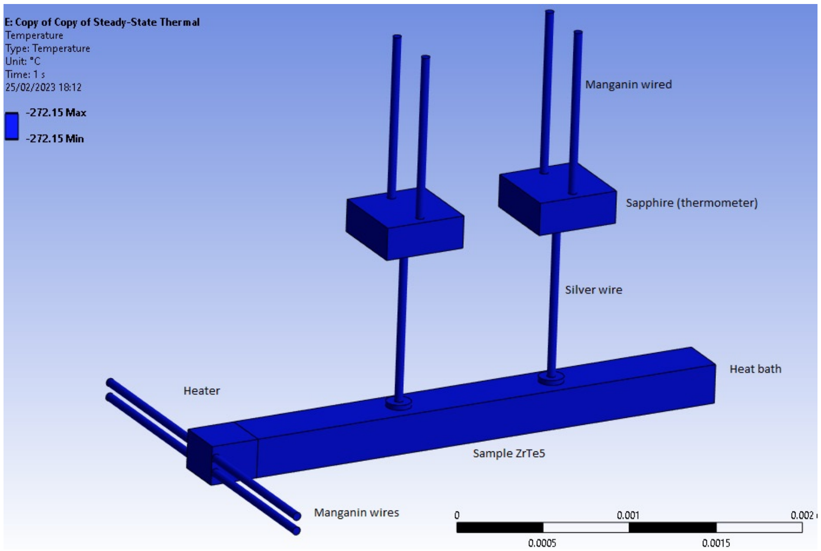

Figure 4. Calculated temperature distribution across the sample, with no heat applied. Radiative corrections are calculated assuming the sample thermal bath being at 1K and surrounding chamber at 4K

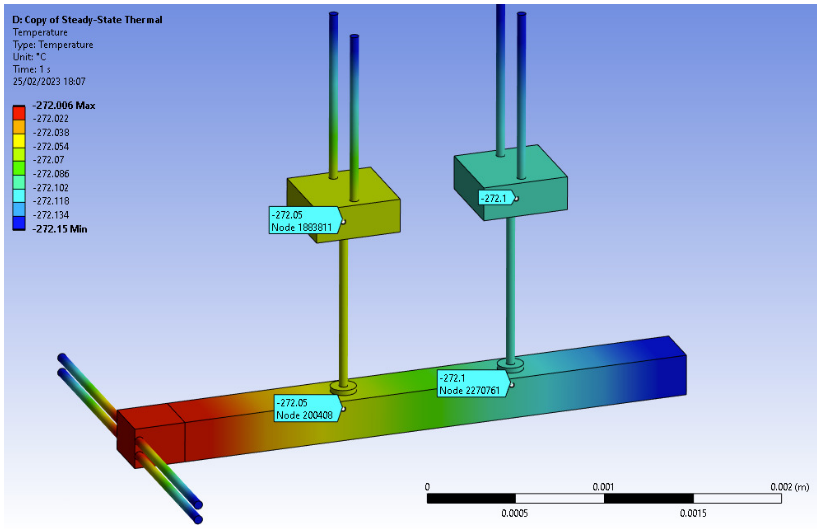

Figure 5. Calculated temperature distribution across the sample, with XX Watt power applied to the heater. Radiative corrections are calculated assuming the sample thermal bath being at 1K and surrounding chamber at 4K
